# Cost-Effectiveness and Clinical Outcomes of Controlled Ovarian Stimulation With Follitropin Delta and Follitropin Alfa: A Retrospective Study

**DOI:** 10.7759/cureus.76371

**Published:** 2024-12-25

**Authors:** Masato Kobanawa, Nanako Iwami, Masachi Hanaoka, Kunihiro Enatsu, Takuhiko Ichiyama

**Affiliations:** 1 Gynecology, Kobanawa Clinic, Ibaraki, JPN; 2 Reproductive Medicine, Kamiya Ladies Clinic, Hokkaido, JPN; 3 Reproductive Medicine, Hanaoka In Vitro Fertilization (IVF) Clinic Shinagawa, Tokyo, JPN; 4 Reproductive Medicine, Hanabusa Women's Clinic, Nishinomiya, JPN; 5 Reproductive Medicine, Torch Clinic, Tokyo, JPN

**Keywords:** cost effectiveness, cumulative live birth rate, follitropin alfa, follitropin delta, incremental cost-effectiveness ratio (icer)

## Abstract

Aim: This study compared the cost-effectiveness of two recombinant follicle-stimulating hormones (rFSH) formulations, Follitropin Delta and Follitropin Alfa, in controlled ovarian stimulation using cumulative live birth rates as an efficacy indicator.

Methodology: This retrospective study was conducted across five clinics in Japan from April 2022 to December 2023, involving 446 first assisted reproductive technology (ART) cycles (200 with Follitropin Delta and 246 with Follitropin Alfa) were treated with rFSH monotherapy using either Follitropin Delta or Follitropin Alfa. We compared clinical outcomes such as cumulative pregnancy and live birth rates and analyzed cost-effectiveness using the cumulative live birth rates as the efficacy indicator and the incremental cost-effectiveness ratio (ICER).

Results: The Follitropin Delta group had a significantly lower incidence of ovarian hyperstimulation syndrome (15.90% vs. 27.00%, *P* = 0.045) and higher cumulative pregnancy rates than the Follitropin Alfa group (87.30% vs. 76.20 %; *P* = 0.03) after propensity score matching (PSM). Although cumulative live birth rates showed no significant differences (85.70% vs. 76.20%, *P* = 0.08) and Follitropin Delta demonstrated higher cost than Follitropin AlfaFollitropin Alfa (832,036 yen and 826,936 yen), ICER indicated low costs per percentage of live births (538.58 yen/%: 95% confidence interval [CI]: 275.34-12,568.69 yen).

Conclusions: Using Follitropin Delta for controlled ovarian stimulation in ART may be more cost-effective than Follitropin Alfa under Japan’s Health Care Insurance System, offering higher cumulative live birth rates and minimal additional costs.

## Introduction

In Japan, Follitropin Delta and Follitropin Alfa are marketed as recombinant follicle-stimulating hormone (rFSH) formulations. Follitropin Delta is an rFSH derived from a human fetal retinal cell line intended for controlled ovarian stimulation (COS) [1]. In contrast, Follitropin Alfa is an rFSH derived from Chinese hamster ovarian (CHO) cells [2]. The more acidic isoform, Follitropin Delta, and the less acidic isoform, Follitropin Alfa, are the same rFSH formulations but differ in their sialic acid content [3]. Assisted reproductive technology (ART) in Japan has been covered by the healthcare insurance system since April 2022. Japan has a universal health insurance system covering 70% of ART costs, so most patients pay only 30% of the treatment costs. Both Follitropin Delta and Follitropin Alfa are pen-type, self-injection drugs, and insurance covers home self-injection guidance and management. COS, mainly through self-injection, reduces the number of hospital visits, thereby increasing patient satisfaction and reducing overcrowding at clinics, benefiting both patients and clinics. Therefore, the demand for both products is expected to increase because ART is now covered by insurance.

The Japanese Healthcare Insurance System for ART includes additional points for oocyte retrieval, fertilization, embryo culture, and embryo freezing. Consequently, the number of oocytes retrieved and embryos available for transfer has become important from both medical and economic standpoints.

Recently, cumulative pregnancy and live birth rates per oocyte retrieval cycle have attracted worldwide attention as key performance indicators of ART [4, 5]. Important factors influencing cumulative pregnancy and live birth rates include the number of oocytes retrieved and the number of embryos available for transfer [6-9]. However, the greater the number of oocytes retrieved and the greater the number of blastocysts, the higher the cost, which may be to the detriment of the patient. This is because the higher the dose of FSH administered, the greater the number of oocytes retrieved, but the more drugs used, the higher the cost [10-12]. Moreover, in Insurance Scores for Assisted Reproductive Technology in Japan, the number of eggs retrieved, embryos frozen, and embryos transferred affect the cost, as the amount is added as each number is increased.

Therefore, we need to be conscious of cost-effectiveness in selecting treatments in clinical practice.

This multicenter study compared the cumulative live birth rate as a clinical outcome to compare the efficacy of two rFSH formulations, Follitropin Delta and Follitropin Alfa. We also compared the costs required for live birth, and compared the cost-effectiveness using the cumulative birth rate as an effectiveness indicator.

## Materials and methods

A total of 446 cycles (200 cycles in the Follitropin Delta group and 246 cycles in the Follitropin Alfa group) meeting the criteria were retrospectively studied at five centers that performed the first ART covered by insurance between April 2022 and December 2023. After approval by the Medical Corporation Kobanawa Clinic Ethic Screening Committee, this study was conducted with opt-out disclosure of information.

This study included treatment-naive women with COS who were treated with rFSH monotherapy using either Follitropin Delta or Follitropin Alfa. Eligible patients were those covered by the Healthcare Insurance System and aged up to 42 years.

**Figure 1:**
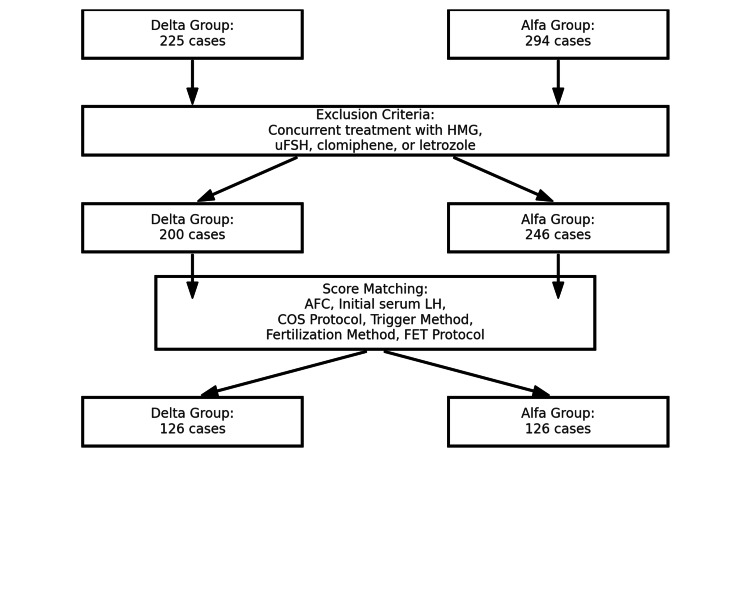
Flowchart of patient selection and score matching process. This flowchart illustrates the patient selection process and score matching for Delta and Alfa groups in a clinical study. This flowchart highlights the systematic approach to ensure balanced comparison between the two groups. Image credit: Masato Kobanawa HMG, human menopausal gonadotropin; uFSH, urinary follicle-stimulating hormone; AMH, anti-Müllerian hormone; AFC, antral follicle count; COS protocol, controlled ovarian stimulation protocol; FET protocol, frozen embryo transfer protocol

Eligible patients underwent their first COS for ART using either Follitropin Alfa or Follitropin Delta. Exclusion criteria included patients who deviated from the prescription guidelines for Follitropin Delta or Alfa, and those who received concurrent concomitant treatment with human menopausal gonadotropin (HMG), urinary FSH (uFSH), clomiphene, or letrozole (Figure 1).

Propensity score matching (PSM) was performed to balance baseline characteristics between the Delta (200 cases) and Alfa (246 cases) groups. In this study, 1:1 nearest neighbor PSM without replacement was performed to create balanced cohorts. A caliper of 0.2 standard deviations of the logit-transformed propensity score was applied to minimize the risk of poor matches, ensuring that only cases with sufficiently similar propensity scores were paired. Matching was based on key covariates calculated using logistic regression, including antral follicle count (AFC), initial serum LH level, COS protocol, trigger method, fertilization method, and frozen embryo transfer (FET) protocol. After matching, 126 cases were retained in both groups, with 63% of the Delta group (126/200) and 51% of the Alfa group (126/246) successfully matched, ensuring comparable cohorts for subsequent analysis (Figure 1).

At each clinic, daily subcutaneous injections of Follitropin Alfa (Gonal F; Merck BioPharma, Tokyo, Japan) or Follitropin Delta (Rekovelle; Ferring Pharma, Tokyo) as a mono protocol with rFSH were administered starting on days 1-3 of menstruation, using the COS with GnRH antagonist protocol, progestin-primed ovarian stimulation (PPOS), or GnRH agonist short protocol.

The daily individualized dose of Follitropin Delta (Delta group) was determined using the serum AMH level within the previous 12 months and body weight, with a fixed dose used throughout the stimulation [13].

The starting dose of Follitropin Alfa (Alfa group) was administered at daily doses of 150, 225, or 300 IU according to the clinician’s empirical judgment for the first six days. Subsequently, the dose was adjusted by 75 IU according to the individual response during stimulation, with a maximum daily dose of 300 IU.

In the GnRH antagonist protocol, when the primary follicle reached approximately 14 mm, treatment with a GnRH antagonist (Ganirest; MSD, Tokyo) at a dose of 0.25 mg/day was initiated and continued until the triggering day. When several leading follicles reached 18-20 mm, 250 μg of choriogonadotropin alfa (Ovidrel; Merck Biopharma, Tokyo) or 600 μg GnRH agonist (Suprecur; Clinigen, Tokyo) was administered as a trigger.

In the PPOS protocol, patients were administered 10 mg medroxyprogesterone acetate (MPA) or 20 mg dydrogesterone daily from days 1 to 3 of menstruation until the triggering day. When several leading follicles reached 18-20 mm, 250 μg of choriogonadotropin alfa or 600 μg GnRH agonist was administered as a trigger.

In the GnRH agonist short protocol, 900 μg GnRH agonist was administered from days 1 to 3 of menstruation until the triggering day. When several leading follicles reached 18-20 mm, 250 μg of choriogonadotropin alfa was administered as a trigger. In all three protocols, oocyte retrieval was performed 34-36 hours after triggering.

After oocyte retrieval, insemination and intracytoplasmic sperm injection (ICSI) were performed per each clinic’s standards, and the fertilized oocytes were cultured to blastocysts, which were then frozen. Thawed embryos were transferred during the next menstrual cycle or later by hormone replacement cycles (HRCs) or natural cycles (NCs). In HRC, hormone replacement of estrogen (Estrana Tapes, Hisamitsu Pharmaceutical, Tokyo) at 0.72 mg ×4 every other day was started on days 1-3 of menstruation. With endometrial thickening confirmed to be at least 7 mm, progesterone (Lutinus Vaginal Tablets; Ferring Pharma, Tokyo) 100 mg ×3/day was commenced, and blastocyst transfer was performed six days later (P+5). We performed a single embryo transfer (SET). In the NC group, blastocysts were transferred on the fifth day after natural ovulation without the use of drugs. Clinical pregnancy was defined as a case in which the fetal sac was confirmed by transvaginal ultrasonography within four to five weeks of gestation determined from the day of embryo transfer. After pregnancy, birth outcomes were tracked based on reports from the patients or the hospitals where the delivery occurred.

The presence of symptoms such as abdominal distension (Golan classification grade 1 or higher) defined the onset of ovarian hyperstimulation syndrome (OHSS) [14].

The fertilization rate was defined as the number of pronuclei confirmed per insemination by IVF or ICSI punctures. The embryo culture results were compared based on blastocyst rates, defined as the number of high-quality blastocysts (Gardner’s classification [15] of 3BB or higher) per cultured embryo.

The embryo transfer outcomes were compared by clinical pregnancy rate at the first transfer, cumulative pregnancy rate per cycle, and cumulative live birth rate.

Clinical pregnancy was defined as confirmation of the fetal sac by transvaginal ultrasonography at four to five weeks of gestation, measured from the embryo transfer day.

Cumulative pregnancy and live birth rates were calculated as the probability [4] of a single-cycle embryo leading to pregnancy or live birth over multiple transfers.

As the purpose was to compare the results per cycle for each group, the calculation included cycles in which at least one pregnancy and delivery occurred after multiple thawed embryo transfers of frozen embryos per cycle, cycles in which pregnancy did not occur even if all embryos were used, and cycles in which COS was performed but embryo freezing was unsuccessful.

**Figure 2:**
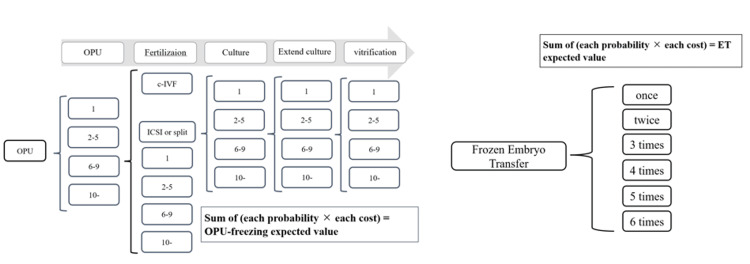
Decision tree: OPU freezing and frozen embryo transfer. The decision tree illustrates the number of components in each process of assisted reproductive treatment under the Japanese insurance system, along with the method for calculating the expected values. Image credit: Masato Kobanawa OPU, oocyte pick-up; ET, embryo transfer; ICSI, intracytoplasmic sperm injection; c-IVF, conventional in vitro fertilization

The cost of COS was first calculated to examine cost-effectiveness by multiplying each cycle’s total gonadotropin dose by the drug price per IU or per μg of each group (price as of December 2023), adding the cost of luteinizing hormone (LH) surge prevention drugs (Suprecur, Ganirest, 10 mg of MPA or 20 mg of dydrogesterone), and including the price of the trigger drug (Ovidrel or Suprecur). The median was used to compare the data of the two groups since the data were non-normally distributed per the Shapiro-Wilk normality test. Next, a decision tree model was created for the number of oocytes retrieved, fertilization method, ICSI, fertilized oocytes, blastocyst culture, embryo freezing (vitrification), and insurance coverage (Figure 2). The probability of transition to each item (Appendices A-D) and insurance coverage ×10 (Appendix E) was applied to calculate the expected value for both formulations. Additionally, the expected values were calculated by multiplying the probability of each embryo transfer by the number of embryo transfers (Appendices B, D) and the median cost per transfer for each group, then adding them together. Since the cost of embryo transfer was also non-normally distributed per the Shapiro-Wilk test, the median was used to compare the data of the two groups. Additionally, the total cost of other medical services (Appendices F-G) was added to the COS cost comparison. The cost from pregnancy to delivery was estimated at 100,000 yen for pregnancy health checkups, based on the Ministry of Health, Labor, and Welfare’s (MHLW) survey on public cost sharing of pregnancy checkups of April 2022 [16]. Delivery costs were set at approximately 482,000 yen, as calculated by the MHLW from the 2022 direct payment system exclusive claims for normal deliveries [17]. The patient-paid amount, set at 30% of the calculated expected cost from COS to embryo transfer [18], along with the total cost of antenatal checkups and delivery, was compared with the cost-effectiveness of clinical outcomes using the incremental cost-effectiveness ratio (ICER) [19]. In this study, the primary endpoint was to compare the cost-effectiveness using ICER calculations with cumulative live birth rate as the effectiveness indicator.

The secondary endpoint was to compare the clinical outcomes of the two groups.

To address uncertainty in this study, the bootstrap method was applied [20] to estimate ICER changes by varying cumulative live birth rate differences between the Delta and Alfa groups within a 95% confidence interval (CI), as variation in cumulative live birth rates may impact ICER.

In this study, the High-Cost Medical Expense Benefit [21] was used to set the willingness-to-pay (WTP) threshold for the ICER. The High-Cost Medical Expense Benefit is a system that reimburses amounts exceeding a designated copayment limit when monthly medical expenses become too high [21]. ART patients frequently use this system, with the maximum threshold set at 24,600 yen for WTP to make a more rigorous comparison with ICER [21]. Furthermore, to illustrate how changes in the WTP threshold affect cost-effectiveness, we created a cost-effectiveness acceptability curve (CEAC). CEAC shows the probability that an intervention is cost-effective across a range of WTP thresholds, based on probabilistic sensitivity analysis. By plotting these probabilities against different thresholds, the CEAC highlights the relationship between WTP and the likelihood of cost-effectiveness. This provides a clear visual representation of how cost-effectiveness conclusions vary with changes in WTP, offering valuable insights for decision-making.

Statistical analysis methods used to compare patient background data were the Mann-Whitney U and chi-square tests. The median was used to compare the two groups since the patient background data were also non-normally distributed according to the Shapiro-Wilk normality test. The chi-square test was used for univariate analysis of the incidence of OHSS, percentage of HRCs, live birth rate at the first embryo transfer, cumulative pregnancy rate, and cumulative live birth rate. Furthermore, multiple logistic regression analyses were performed with adjustment factors. All statistical analyses were performed using EZR (Saitama Medical Center, Jichi Medical University, Saitama, Japan), a graphical user interface for R (R Foundation for Statistical Computing, Vienna, Austria). More precisely, it is a modified version of the R commander designed to add statistical functions frequently used in biostatistics.

## Results

Regarding patient background, significant differences between the two groups were observed in AFC, initial serum LH levels, COS protocols, trigger methods, fertilization methods, and FET protocols (percentage of HRC) (Table 1).

**Table 1:** Patient characteristics before and after propensity score matching (PSM). Data are presented as median [min, max] or % (*n*). Statistical analysis methods used to compare patient background data were the Mann-Whitney U and chi-square tests, and a significant difference was judged to exist when *P* < 0.05. PSM, propensity score matching; COS, controlled ovarian stimulation; AMH, anti-Müllerian hormone; AFC, antral follicle count; E2, estradiol; LH, luteinizing hormone; FSH, follicle-stimulating hormone; PPOS, progestin-primed ovarian stimulation; hCG, human chorionic gonadotropin; GnRH, gonadotropin-releasing hormone; IVF, in vitro fertilization; ICSI, intracytoplasmic sperm injection

Variables	Before PSM			After PSM		*P*-value
	Delta group	Alfa group		Delta group	Alfa group	
	*n* = 200	*n* = 246	*P*-value	*n* = 126	*n* = 126	
Age (years)	35.00 [24.00, 42.00]	35.0 [23.00, 43.00]	0.65	35.00 [24.00, 42.00]	35.00 [23.00, 43.00]	0.47
Gravidity, times	0.00 [0.00, 6.00]	0.0 [0.00, 7.00]	0.77	0.00 [0.00, 6.00]	0.00 [0.00, 7.00]	0.55
Parity, times	0.00 [0.00, 2.00]	0.00 [0.00, 3.00]	0.33	0.00 [0.00, 2.00]	0.00 [0.00, 2.00]	0.29
Period of infertility (years)	1.00 [0.00, 6.00]	1.00 [0.00, 8.00]	0.00	1.00 [0.00, 6.00]	1.00 [0.00, 8.00]	0.01
Body weight (kg)	53.00 [37.00, 94.80]	52.65 [38.00, 106.20]	0.69	53.40 [37.00, 94.80]	54.00 [41.00, 106.20]	0.40
AMH (ng/mL)	3.33 [0.59, 19.3]	2.72 [0.16, 22.96]	0.11	3.40 [0.59, 15.53]	2.66 [0.35, 17.71]	0.21
AFC, follicles	9.00 [0.00, 30.00]	10.00 [0.00, 76.00]	0.0003	9.00 [2.00, 25.00]	9.00 [0.00, 27.00]	0.31
Initial serum E2 (pg/mL)	28.44 [5.00, 96.87]	30.00 [5.00, 100.00]	0.06	28.44 [5.00, 96.87]	30.00 [5.00, 98.79]	0.17
Initial serum LH (IU/L)	5.65 [0.10, 17.10]	5.80 [0.10, 15.75]	0.01	5.80 [0.10, 11.10]	5.80 [0.10, 15.75]	0.40
Initial serum FSH (IU/L)	7.22 [0.30, 21.70]	7.41 [0.30, 20.94]	0.72	7.15 [0.30, 21.70]	7.64 [0.30, 20.94]	0.17
COS protocol, % (*n*)			0.005			1.00
Agonist	6.00 (12)	1.22 (3)		0.79 (1)	1.59 (2)	
Antagonist	79.00 (158)	85.77 (211)		83.34 (105)	78.57 (99)	
PPOS	15.00 (30)	13.41 (33)		15.87 (20)	19.84 (25)	
Trigger method, % (*n*)			0.001			1.00
ｈCG	7.50 (15)	18.29 (45)		3.17 (4)	3.17 (4)	
GnRH agonist	54.00 (108)	67.89 (167)		72.22 (91)	72.22 (91)	
Dual trigger	38.50 (77)	13.82 (34)		24.61 (31)	24.61 (31)	
Fertilization method, % (*n*)			0.001			1.00
Conventional IVF	42.50 (85)	41.06 (101)		57.93 (73)	57.93 (73)	
ICSI	31.50 (63)	19.10 (47)		25.40 (32)	25.40 (32)	
Split	26.00 (52)	39.84 (98)		16.67 (21)	16.67 (21)	

Regarding clinical outcomes, multivariate analysis of the original data showed that the incidence of OHSS was significantly lower in the Delta group than in the Alfa group (14.00% vs. 34.15%; *P* = 0.000749). The total gonadotropin dose was also significantly lower in the Delta and Alfa groups, with 90.00 mcg vs. 132.00 mcg (*P* = 0.000004). The use of the HRC as an embryo transfer protocol was significantly higher in the Delta group (91.38% vs. 78.77%; *P* = 0.01). The cumulative pregnancy rate was 86.50% vs. 84.55% (*P* = 0.85) in univariate analysis, with no difference between the two groups. However, the odds ratio calculated by logistic regression analysis adjusted for confounding factors was 2.83 (95% CI 1.50-5.33). Thus, the statistical cumulative pregnancy rate was 93.93% vs. 84.55% (*P *= 0.001), significantly higher in the Delta group. Similarly, the actual data for the cumulative live birth rate was 78.00% vs. 78.86% (*P *= 0.92), with no difference between the two groups. However, the odds ratio calculated by logistic regression adjusted for confounding factors was 2.27 (95% CI 1.26-4.10). Thus, the cumulative statistical live birth rate was 89.44% vs. 78.86% (*P *= 0.01), significantly higher in the Delta group (Table 2).

**Table 2:** Comparison of clinical outcomes between Follitropin Delta and Follitropin Alfa before propensity score matching. Data were presented as median [min, max] or % (*n*). Two groups were compared by the Mann-Whitney U and chi-square tests, and a significant difference was judged to exist when *P* < 0.05. Multiple regression and logistic regression analyses were also performed with adjustment factors (AFC, initial serum LH level, COS protocol, trigger method, fertilization method, and FET protocol). E2, estradiol; P4, progesterone; OHSS, ovarian hyperstimulation syndrome; HRC, hormone replacement cycle; FET, frozen embryo transfer; COS, controlled ovarian stimulation; AFC, antral follicle count; LH, luteinizing hormone

Variables	Delta group	Alfa group	Univariate *P*-value	Multivariate *P*-value
	*n* = 200	*n* = 246		
Serum E2 on trigger day (pg/mL)	3049.05 [370.10, 8286.00]	2351.50 [ 107.30, 10278.00]	0.88	0.64
Serum P4 on trigger day (pg/mL)	1.00 [ 0.08, 7.57]	0.97 [ 0.05, 10.92]	0.02	0.80
Duration of stimulation (days)	11.00 [6.00, 27.00]	11.00 [2.00, 22.00]	0.41	0.68
Total gonadotropin dose (mcg)	90.00 [42.00, 206.82]	132.00 [22.00, 484.00]	0.000	0.000
Number of oocytes retrieved, oocytes	11.00 [0.00, 44.00]	14.00 [2.00, 48.00]	0.003	0.30
Number of mature oocytes retrieved, oocytes	6.00 [0.00, 23.00]	8.00 [0.00, 22.00]	0.09	0.94
Number of fertilized oocytes, oocytes	7.00 [0.00, 30.00]	9.00 [1.00, 31.00]	0.001	0.32
Fertilization rate (%)	66.70 [0.00, 100.00]	66.7 [6.25, 100.0]	0.07	0.13
Number of good-quality blastocysts, blastocysts	3.00 [0.00, 19.00]	4.00 [1.00, 16.00]	0.001	0.54
Blastocyst Quality Score (points)	36.00 [0.00, 45.00]	36.00 [1.00, 45.00]	0.03	0.22
Blastocyst rate (%)	50.00 [0.00, 100.00]	50.00 [6.25, 100.00]	0.73	0.57
Incidence of OHSS, *n* (%)	14.00 (28.00)	34.15 (84.00)	0.000002	0.001
Total number of embryo transfers, times	1.00 [0.00, 5.00]	1.00 [1.00, 6.00]	0.45	0.76
Percentage of HRC, % (*n*)	91.38 (182)	78.77 (194)	0.003	0.01
Live birth rate at first embryo transfer, % (*n*)	51.50 (103.00)	63.01 (155.00)	0.02	0.14
Cumulative pregnancy rate, % (*n*)	86.50 (173.00)	84.55 (208.00)	0.85	0.001
Cumulative live birth rate, % (*n*)	78.00 (156.00)	78.86 (194.00)	0.92	0.01

PSM of the Delta group (*n* = 126) vs. the Alfa group (*n* = 126) showed that the incidence of OHSS was significantly lower in the Delta group (15.90% vs. 27.00%, *p* = 0.045). The total gonadotropin dose was also significantly lower in the Delta and Alfa groups, with 91.00 mcg vs. 181.50 mcg (*P *= 0.0002). The cumulative pregnancy rate was significantly higher in the Delta group (87.30% vs. 76.20 %; *P* = 0.03). The cumulative live birth rate was 85.70% vs. 76.20% (*P* = 0.08), which tended to be higher in the Delta group (Table 3).

**Table 3:** Comparison of clinical outcomes between Follitropin Delta and Follitropin Alfa after propensity score matching. Data were presented as median [min, max] or % (*n*). Two groups were compared by Mann-Whitney U and chi-square tests, and a significant difference was judged to exist when *P* < 0.05. E2, estradiol; P4, progesterone; OHSS, ovarian hyperstimulation syndrome; HRC, hormone replacement cycle

variables	Delta group	Alfa group	
	*n *= 126	*n* = 126	*P*-value
Serum E2 on trigger day (pg/mL)	3300.00 [690.20, 7963.0]	3131.00 [139.30, 10278.00]	0.53
Serum P4 on trigger day (pg/mL)	1.01 [0.18, 4.97]	1.00 [0.11, 10.92]	0.59
Duration of stimulation (days)	11.50 [6.00, 27.00]	12.00 [7.00, 22.00]	0.05
Total gonadotropin dose (mcg)	91.00 [42.00, 206.82]	181.50 [66.00, 396.00]	0.000
Number of oocytes retrieved, oocytes	12.00 [2.00, 44.00]	14.00 [3.00, 38.00]	0.28
Number of mature oocytes retrieved, oocytes	7.00 [0.00, 22.00]	8.50 [0.00, 22.00]	0.30
Number of fertilized oocytes, oocytes	7.00 [1.00, 30.00]	9.00 [1.00, 29.00]	0.23
Fertilization rate (%)	67.00 [9.00, 100.00]	67.00 [23.00, 100.00]	0.82
Number of good-quality blastocysts, blastocysts	4.00 [1.00, 19.00]	4.00 [1.00, 16.00]	0.39
Blastocyst rate (%)	51.67 [11.11, 100.00]	50.00 [6.25, 100.00]	0.62
Blastocyst Quality Score (points)	36.00 [8.00, 45.00]	36.00 [8.00, 45.00]	0.24
Incidence of OHSS, % (*n*)	15.90 (20.00)	27.00 (34.00)	0.045
Total number of embryo transfers, times	2.00 [1.00, 5.00]	1.00 [1.00, 6.00]	0.17
Percentage of HRC, % (*n*)	100 (126)	100 (126)	0.46
Live birth rate at first embryo transfer, % (*n*)	52.40 (66.00)	62.70 (79.00)	0.13
Cumulative pregnancy rate, % (*n*)	87.30 (110.00)	76.20 (96.00)	0.03
Cumulative live birth rate, % (*n*)	85.70 (108.00)	76.20 (96.00)	0.08

**Figure 3:**
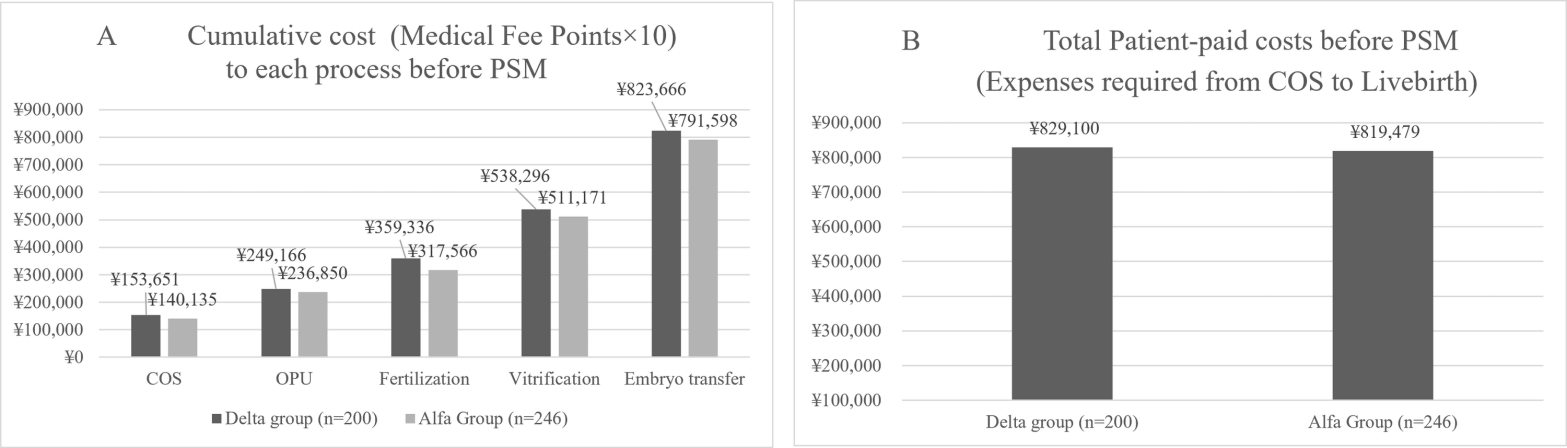
Comparison of cumulative cost to each process between two groups before PSM. (A) Comparison of cumulative cost between the two groups before PSM. Each process is from COS to OPU, fertilization, vitrification, embryo transfer, and live birth. The cumulative cost is the sum of the expected values of each process. (B) The bar graph illustrates the total expenses incurred by patients from COS to live births in two groups. PSM, propensity score matching; COS, controlled ovarian stimulation; OPU, oocyte pick-up

In the cost-effectiveness analysis, the median cost of COS for all data was 153,651 yen in the Delta group and 140,135 yen in the Alfa group. The costs of oocyte retrieval were 249,166 and 236,850 yen, respectively, while the costs of embryo freezing were 538,296 and 511,171 yen, respectively. The costs of cumulative embryo transfer were 823,666 yen and 791,598 yen, respectively. The projected patient-paid costs of delivering a baby using cumulative embryo transfer were 829,100 and 819,479 yen, respectively (Figure 3).

**Figure 4:**
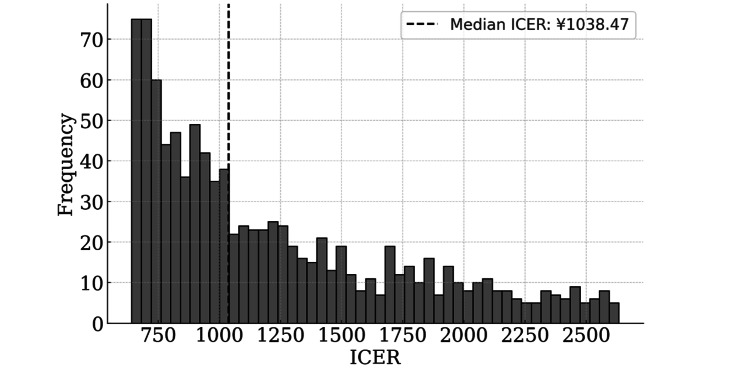
Histogram of ICER before PSM, calculated using the bootstrap method. This graph shows the distribution of ICER for different cumulative live birth rate differences. The ICER represents the cost per 1% increase in live birth rates. A total of 1,000 bootstrap samples were generated, with live birth rate differences ranging from 3.6% to 15.0% (95% confidence interval [CI]) and a fixed cost difference of 9,620 yen. The dashed line indicates the median ICER value. ICER, incremental cost-effectiveness ratio

The ICER for the cumulative live birth rate as an effectiveness measure was based on the multivariate statistical cumulative live birth rate of 89.44% vs. 78.86%, with an odds ratio of 2.27 (95% CI 1.26-4.10). Since the cumulative live birth rate difference between the two groups (Delta group - Alfa group) varies from 3.60% to 15.00%, using the bootstrap method, the median ICER was 1,038.47 yen/% (95% CI: 656.51-2,473.39 yen). Assuming that the amount of WTP for ICER (the threshold of ICER) was 24,600 yen [21], the value of ICER would be lower than this. The Delta group was considered cost-effective because the ICER value was less than this amount (Figure 4).

**Figure 5:**
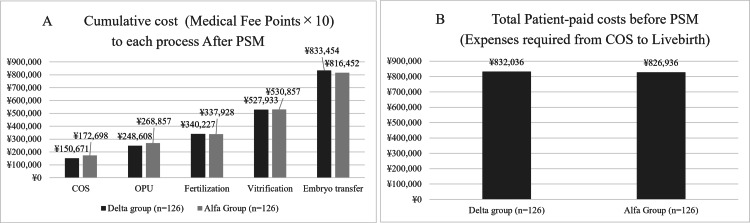
Comparison of cumulative cost to each process between two groups after PSM. (A) This graph shows the comparison of cumulative cost between the two groups after PSM. Each process is from COS to OPU, fertilization, vitrification, embryo transfer, and live birth. The cumulative cost is the sum of the expected values of each process. (B) This bar graph illustrates the total expenses incurred by patients from COS to live births in two groups. PSM, propensity score matching; COS, controlled ovarian stimulation; OPU, oocyte pick-up

In the patient population for which PSM was performed, the median costs of COS were 150,671 yen for the Delta group and 172,698 yen for the Alfa group, respectively. The costs of oocyte retrieval were 248,608 and 268,857 yen, respectively. The costs of embryo freezing were 527,933 and 530,857 yen, respectively. The costs of cumulative embryo transfer were 833,454 and 816,452 yen, respectively. The projected patient-paid costs of delivering a baby using cumulative embryo transfer were 832,036 and 826,936 yen, respectively (Figure 5).

**Figure 6:**
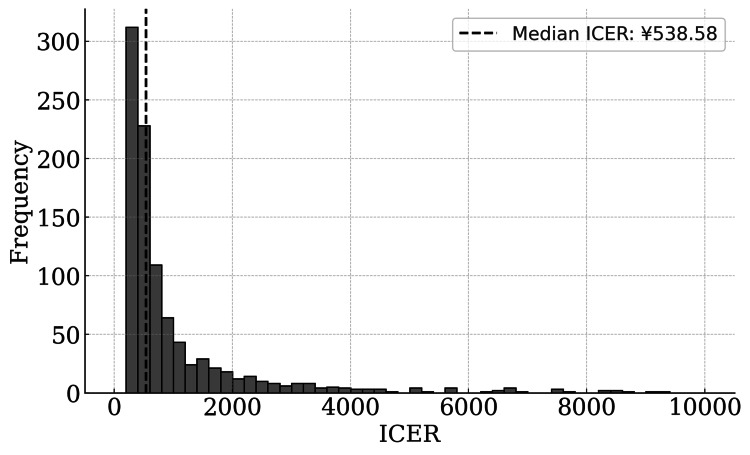
Histogram of ICER after PSM, calculated using the bootstrap method. This graph displays the distribution of ICER for different cumulative live birth rate differences based on a bootstrap analysis. The ICER measures the cost per 1% increase in live birth rates. A total of 1,000 bootstrap samples were generated, with cumulative live birth rate differences ranging from -0.1% to 19.1% (95% confidence interval [CI]) and a fixed cost difference of 5,100 yen. The dashed line represents the median ICER value. ICER, incremental cost-effectiveness ratio; PSM, propensity score matching

The ICER for the cumulative live birth rate as an effectiveness measure was 85.70% vs. 76.20%. The cumulative live birth rate difference between the two groups (Delta group - Alfa group) varied from -0.1% to 19.1% from the 95% CI (*P *= 0.095); the median ICER was 538.58 yen/% (95% CI: 275.34-12,568.69 yen). Assuming that the amount of ICER WTP (the threshold of ICER) was 24,600 yen [21], the Delta group was considered cost-effective because the ICER value was less than this amount (Figure 6).

**Figure 7:**
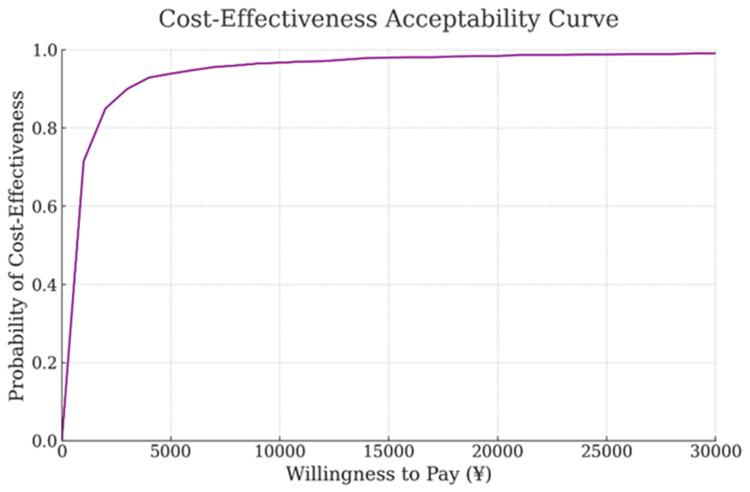
Cost-effectiveness acceptability curve (CEAC). This CEAC shows the probability that the intervention is cost-effective, given a cost difference of 5,100 yen and a cumulative live birth rate difference range of -0.1% to 19.1%. The X-axis represents the willingness-to-pay (WTP) from 0 to 30,000 yen (¥), and the Y-axis shows the probability of cost-effectiveness. The curve rises sharply, reaching nearly 100% around a WTP of 10,000 yen, and then levels off. This suggests that the intervention is highly cost-effective at WTP values above 10,000 yen.

In addition, a CEAC was created to verify whether cost-effectiveness is acceptable assuming that WTP changes. The WTP at which the cost-effectiveness probability reached 95% was 7,000 yen, and when the WTP was 24,600 yen, the cost-effectiveness probability was approximately 98.8% (Figure 7).

## Discussion

This study aims to compare the cost-effectiveness and clinical outcomes of two rFSH formulations, Follitropin Delta and Follitropin Alfa, in COS. The main efficacy indicator used is the cumulative live birth rate, with ICER to assess cost-effectiveness.

FSH is a glycoprotein consisting of two non-covalently linked polypeptide chains, α and β. Follitropin Delta has a glycan structure similar to endogenous FSH, with not only α2,3 sialic acid but also N-acetylgalactosamine (GalNAc) and α2,6 sialic acid, making it the more acidic isoform [1]. Follitropin Alfa has only α2,3-linked sialic acid [22]. In addition, glycoforms with α2,6-linked sialic acid result in slower elimination rates compared to glycoforms with α2,3-linked sialic acid; the slower elimination rate may depend on the clearance mechanism of these different forms. α2,3-linked sialic acid is metabolized mainly by the kidneys, whereas α2,6-linked sialic acid is metabolized primarily by the asialoglycoprotein receptor (ASGPR) in the liver [23]. This difference in sialic acid content results in a lower clearance of Follitropin Delta from the serum, leading to increased exposure and a greater pharmacodynamic response [1]. These differences in isoforms due to sialic acid binding may affect follicle development via COS and hormonal kinetics. Several comparisons between Follitropin Delta and Follitropin Alfa have been reported.

In the STORK trial comparing Follitropin Delta and Follitropin Beta, the total gonadotropin dose was significantly lower in the Follitropin Delta group; however, there were no significant differences in oocyte retrieval, blastocyst numbers, or clinical pregnancy rates [24].

In the ESTHER-1 trial comparing Follitropin Delta with Follitropin Alfa, the total gonadotropin dose tended to be lower, while the number of stimulation days was longer in the Follitropin Delta group [25]. There were no significant differences in the number of retrieved oocytes, blastocysts, or clinical pregnancy rates [25].

In the GRAPE trial, which also compared Follitropin Delta and Follitropin Alfa, the total gonadotropin dose was lower, and the number of stimulation days was significantly longer in the Follitropin Delta group [26]. Oocyte retrieval and the number of early embryos available for transfer were significantly higher in the Follitropin Alfa group [26]. Furthermore, the live birth rate and the live birth rate at four weeks after birth were significantly higher in the Follitropin Delta group [26].

In this study, we compared the efficacy of Follitropin Delta and Follitropin Alfa in COS. Similar to that in a previous report, OHSS and total gonadotropin dose incidences were significantly lower in the Follitropin Delta group [26]. Cumulative pregnancy and live birth rates were higher in the delta group. Additionally, we evaluated the cost-effectiveness of Follitropin Delta and Follitropin Alfa in COS.

A previous cost-effectiveness analysis of our single-center Japanese study on Follitropin Delta and Follitropin Alfa in COS found that Follitropin Delta was more cost-effective than Follitropin Alfa in terms of the number of oocytes retrieved, fertilized oocytes, blastocysts, and clinical pregnancy rate [27]. Although the outcome is important, its cost-effectiveness has also been noted to be important. In particular, it has been suggested that cost-effectiveness should be considered based on the mean or median efficacy value and its cost difference, regardless of whether the difference in efficacy is statistically significant in the insurance setting [28]. Since this previous study was a single-center research effort and adopted only the antagonist protocol of COS, selection bias may have occurred. This study aimed to reduce selection bias, including various COS protocols, and produce results similar to those of the general population as a multicenter study involving facilities in various regions of Japan.

Therefore, cost-effectiveness studies should compare the effect indicators and ICER [19].

There is one other report from another country comparing the cost-effectiveness of Follitropin Delta and Follitropin Alfa. A cost-effectiveness study of Follitropin Delta and Follitropin Alfa using the National Health Service (NHS) in the United Kingdom reported no differences between the two drugs [29]. Follitropin Delta demonstrated comparable costs to Follitropin Alfa in the first fresh IVF cycle, with an average total cost of £5,797-£5,818 compared to £5,809-£5,836 for Follitropin Alfa. It showed notable advantages by reducing OHSS prevention rates (2.36% vs. 4.67%) and OHSS incidence (3.46% vs. 4.84%), contributing to slight cost savings of £2-£26 per cycle. These findings highlight Follitropin Delta as a cost-effective and safer alternative for IVF treatment [29].

Thus, it can be understood that Follitropin Alfa is a cost-effective formulation compared to other formulations, but its cost-effectiveness is unclear compared to that of Follitropin Delta.

In this study, we compared the cost-effectiveness of Follitropin Alfa and Follitropin Delta (rFSH) using the cumulative live birth rate as the effectiveness index. The cumulative live birth rate tended to be higher in the delta group, which may have contributed to the cost-effectiveness analysis. One possible mechanism for this is that the total gonadotropin dose was significantly lower in the delta group than in the Alfa group despite having the same background after PSM, which may have improved the quality of embryonic development in the delta group. This study showed a significant decrease in the number of good-quality blastocysts with a higher total gonadotropin dose in all patients after score-matching (regression analysis, *P* = 0.003). Moreover, the number of good-quality blastocysts per gonadotropin dose (mcg) was significantly higher in the Delta group than in the Alfa group (0.03 vs. 0.02, *P *= 0.0007). A recent report also showed that total gonadotropin levels affect embryo quality [30].

The same report suggested that high levels of exogenous gonadotropins interfere with oocyte meiosis and chromosome segregation, resulting in increased embryonic aneuploidy rates [30].

Gonadotropin levels have also been suggested to cause chromosomal aberrations in oocytes [31]. However, it has been demonstrated that the use of exogenous gonadotropins does not affect embryonic aneuploidy rates [32-34]. Therefore, the possibility that differences in the gonadotropin dose affect the cumulative live birth rate cannot be ruled out, although the exact reason for this is unclear. In Japan, the healthcare insurance system for infertility treatment began in April 2022, and the costs from oocyte retrieval to embryo transfer were set to standardize medical care. In particular, until the embryos were produced, the number of oocytes retrieved, number of mature oocytes (ICSI), number of fertilization and embryo cultures, number of blastocyst cultures, and number of frozen embryos were evaluated on a piece-rate system, where the insurance point addition increased as the numbers increased [35]. Therefore, the number of oocytes retrieved and the number of good blastocysts, such as the number of embryos available for transfer, are important factors from the management viewpoint in insurance treatment because insurance points are added.

In addition, the number of oocytes retrieved and several embryos available for transfer are important factors related to cumulative pregnancy and live birth rates, which are indicators of the achievement of pregnancy and delivery in a single oocyte retrieval [6-9]. Moreover, although the patient’s costs increase as the number of embryos increases, the number of oocytes retrieved and many good blastocysts available for transfer are considered directly related to patient satisfaction as compensation for the patient’s COS, considering the insurance coverage of 30%, decrease in the frequency of complications in oocyte retrieval, and reduction in the time to pregnancy and delivery. The pregnancy rate after embryo transfer is also considered the most important factor in patient satisfaction and the outcome of ART (i.e., COS, oocyte retrieval, and embryo transfer) at the facility. Based on the results of this study, Follitropin Delta is considered a more cost-effective and efficient drug for ART under Japanese insurance coverage from a health economics perspective.

A limitation of this study is that examination costs may vary among clinics; however, as this analysis assesses the cost-effectiveness of COS for live births, oocyte retrieval, embryo freezing, and embryo transfer in each group, a common cost was assumed across clinics. In addition, the study’s sample size was arbitrary, with confirmed birth outcomes over a fixed period at five clinics; thus, the population cannot be completely estimated and is subject to uncertainty. Therefore, considering the method and results of this study, with the protocol and other conditions in place,

Randomized controlled trials (RCTs) with larger sample sizes are needed to compare COS with Follitropin Delta and Follitropin Alfa. Such RCTs provide conclusive evidence to determine which approaches are truly cost-effective. Future efforts should focus on conducting RCTs to further evaluate the cost-effectiveness of these treatments.

 This study is the first multicenter investigation using real-world data to compare the cost-effectiveness of two rFSH (Follitropin Delta and Follitropin Alfa) in ART, taking into account the Japanese Healthcare Insurance System. Under the Japanese insurance system, the use of Follitropin Delta is expected to increase the cumulative live birth rate, with an increase in cost that is less than the willingness to pay compared to that with the use of Follitropin Alfa. In conclusion, Follitropin Delta demonstrated a tendency to be more cost-effective than Follitropin Alfa.

## Conclusions

This study is the first multicenter investigation using real-world data to compare the cost-effectiveness of two rFSH (Follitropin Delta and Follitropin Alfa) in ART, taking into account the Japanese Healthcare Insurance System. Under the Japanese insurance system, the use of Follitropin Delta is expected to increase the cumulative live birth rate, with an increase in cost that is less than the WTP compared to that with the use of Follitropin Alfa. In conclusion, Follitropin Delta demonstrated the potential to be more cost-effective than Follitropin Alfa.

## Appendices

Appendix A 

**Table 4:** Probability for each number in the decision tree model from OPU to vitrification in Original Data: Delta group (n=200) vs. Alfa group (n = 246). This table shows probability of branching to each number in each process in the decision tree.

Category	Group	0	1	2–5	6–9	10-
Number of Oocytes Retrieved	Delta Group	0.50%	0.00%	11.00%	24.50%	64.00%
	Alfa Group	0.00%	0.00%	11.80%	17.90%	70.30%
Number of ICSI Punctures	Delta Group	0.00%	3.20%	30.20%	27.00%	39.70%
	Alfa Group	0.00%	0.00%	21.30%	36.20%	42.60%
ICSI Punctures in Split Culture	Delta Group	1.90%	0.00%	48.10%	36.50%	13.50%
	Alfa Group	0.00%	4.10%	41.80%	41.80%	12.20%
Number of Embryo Cultures	Delta Group	1.00%	2.00%	34.00%	34.50%	28.50%
	Alfa Group	0.00%	1.20%	25.20%	27.60%	45.90%
Number of Blastocyst Cultures	Delta Group	1.00%	1.50%	37.00%	33.00%	27.50%
	Alfa Group	0.00%	1.60%	26.40%	27.20%	44.70%
Number of Frozen Embryos	Delta Group	1.50%	23.50%	50.00%	16.50%	8.50%
	Alfa Group	0.00%	13.40%	52.80%	24.40%	9.30%

Appendix B 

**Table 5:** Probability by number in decision tree model of Number of Embryo Transfers (Original Data). This table shows probability of branching to each number in the decision tree.

Group	0	1	2	3	4	5	6
Delta Group	1.50%	49.00%	34.50%	8.50%	5.50%	1.00%	0.00%
Alfa Group	0.00%	57.70%	23.20%	14.60%	3.30%	0.00%	0.80%

Appendix C 

**Table 6:** Probability by number in decision tree model from OPU to vitrification in Propensity Score Matching (PSM) data: delta group (n = 126) vs. alfa group (n = 126). This table shows probability of branching to each number in each process in the decision tree.

Category	Group	0	1	2–5	6–9	10+
Number of Oocytes Retrieved	Delta Group	0.00%	0.00%	6.30%	22.20%	71.40%
	Alfa Group	0.00%	0.00%	14.30%	15.90%	69.80%
Number of ICSI Punctures	Delta Group	0.00%	0.00%	12.50%	34.40%	53.10%
	Alfa Group	0.00%	0.00%	28.10%	28.10%	43.80%
ICSI Punctures in Split Culture	Delta Group	4.80%	0.00%	38.10%	42.90%	14.30%
	Alfa Group	0.00%	0.00%	47.60%	33.30%	19.00%
Number of Embryo Cultures	Delta Group	0.00%	1.60%	27.00%	35.70%	35.70%
	Alfa Group	0.00%	0.80%	27.80%	27.00%	44.40%
Number of Blastocyst Cultures	Delta Group	0.00%	0.80%	31.00%	34.10%	34.10%
	Alfa Group	0.00%	1.60%	28.60%	27.00%	42.90%
Number of Frozen Embryos	Delta Group	0.00%	20.60%	48.40%	22.20%	8.70%
	Alfa Group	0.00%	15.10%	50.80%	23.00%	11.10%
Number of Embryo Transfers	Delta Group	0.00%	46.80%	34.90%	8.70%	7.90%
	Alfa Group	0.00%	57.90%	22.20%	15.10%	2.40%

Appendix D 

**Table 7:** Probability by number in decision tree model of Number of Embryo Transfers (PSM Data). This table shows probability of branching to each number in the decision tree.

Group	0	1	2	3	4	5	6
Delta Group	0.00%	46.80%	34.90%	8.70%	7.90%	1.60%	0.00%
Alfa Group	0.00%	57.90%	22.20%	15.10%	2.40%	0.00%	1.60%

Appendix E 

**Table 8:** Insurance scores for assisted reproductive technology in Japan *10. This table shows the costs at each step.

Category	Sub-category (each numerical value)	Cost (JPY)
Oocyte Retrieval	Oocyte Pick-up (any number)	¥32,000
	Number of Oocytes (1)	¥24,000
	Number of Oocytes (2–5)	¥36,000
	Number of Oocytes (6–9)	¥55,000
	Number of Oocytes (10+)	¥72,000
Conventional IVF	IVF Procedure (any number)	¥42,000
ICSI Punctures	Number of ICSI Punctures (1)	¥48,000
	Number of ICSI Punctures (2–5)	¥68,000
	Number of ICSI Punctures (6–9)	¥100,000
	Number of ICSI Punctures (10+)	¥128,000
Split Culture (ICSI)	Number of Split Culture (1)	¥69,000
	Number of Split Culture (2–5)	¥89,000
	Number of Split Culture (6–9)	¥121,000
	Number of Split Culture (10+)	¥149,000
Embryo Culture	Number of Embryo Cultures (1)	¥45,000
	Number of Embryo Cultures (2–5)	¥60,000
	Number of Embryo Cultures (6–9)	¥84,000
	Number of Embryo Cultures (10+)	¥105,000
Blastocyst Culture	Number of Blastocyst Cultures (1)	¥15,000
	Number of Blastocyst Cultures (2–5)	¥20,000
	Number of Blastocyst Cultures (6–9)	¥25,000
	Number of Blastocyst Cultures (10+)	¥30,000
Frozen Embryos	Number of Frozen Embryos (1)	¥50,000
	Number of Frozen Embryos (2–5)	¥70,000
	Number of Frozen Embryos (6–9)	¥102,000
	Number of Frozen Embryos (10+)	¥130,000

Appendix F 

**Table 9:** Drug prices in 2022-2023.

Item	Cost (JPY)
Follitropin Delta (Rekovelle Subcutaneous 72μg Pen)	¥45,582
Follitropin Alfa (Gonal-F Subcutaneous Pen 900)	¥30,389
GnRH Antagonist (Ganirest Subcutaneous 0.25mg Syringe)	¥8,904
GnRH Agonist (Suprecur Nasal Spray 0.15%)	¥7,122
Recombinant hCG (Ovidrel Subcutaneous Syringe 250μg)	¥2,906
Intravenous Anesthetic (Propofol Intravenous Injection 1% 20mL)	¥501
Analgesic (Sosegon Injection 15mg)	¥59
Antibiotic (Cefotiam Intravenous 1g Bag)	¥797
Infusion Solution (Solacet F Infusion)	¥189

Appendix G 

**Table 10:** Consultation fees in 2022-2023.

Service	Cost (JPY)
Follow-up Consultation + Itemized Receipt Fee	¥740
Transvaginal Ultrasound	¥5,300
Comprehensive Hormone Blood Test	¥4,100
Assisted Reproductive Technology Management Fee	¥3,000
Home Self-Injection Management Fee	¥6,500
Initial Introduction Fee	¥5,800
Needle Fee	¥1,300

Appendix H

**Table 11:** Surgery fees in 2022-2023.

Procedure	Cost (JPY)
Oocyte Pick-up	¥32,000
Intravenous Anesthesia (more than 10 minutes)	¥6,000
Respiratory and Cardiac Monitoring Fee	¥500
Noninvasive Arterial Oxygen Saturation Monitoring	¥300
Noninvasive Continuous Blood Pressure Monitoring	¥1,000
Oxygen Usage (Large Oxygen Cylinder)	¥0.42 / L
